# Optoelectronic system for the determination of blood volume in pneumatic heart assist devices

**DOI:** 10.1186/s12938-015-0111-8

**Published:** 2015-12-10

**Authors:** Grzegorz Konieczny, Tadeusz Pustelny, Maciej Setkiewicz, Maciej Gawlikowski

**Affiliations:** Department of Optoelectronics, Silesian University of Technology, 2A Akademicka Str., 44-100 Gliwice, Poland; Foundation of Cardiac Surgery Development Zabrze, 345a Wolności Str., 41-800 Zabrze, Poland

**Keywords:** Ventricular assist device, Reflectance measurements, Transient blood volume, Cardiac output, Optoelectronic sensor

## Abstract

**Background:**

The following article describes the concept of optical measurement of blood volume in ventricular assist devices (VAD’s) of the pulsatile type. The paper presents the current state of art in blood volume measurements of such devices and introduces a newly developed solution in the optic domain. The objective of the research is to overcome the disadvantages of the previously developed acoustic method—the requirement of additional sensor chamber.

**Methods:**

The idea of a compact measurement system has been introduced, followed by laboratory measurements. Static tests of the system have been presented, followed by dynamic measurements on a physical model of the human ventricular system. The results involving the measurements of blood chamber volume acquired by means of an optical system have been compared with the results acquired by means of the Transonic T410 ultrasound flow rate sensor (11PLX transducer, uncertainty ±5 %).

**Results:**

Preliminary dynamic measurements conducted on the physical model of the human cardiovascular system show that the proposed optical measurement system may be used to measure the transient blood chamber volumes of pulsatile VAD’s with the uncertainties (standard mean deviation) lower than 10 %.

**Conclusions:**

The results show that the noninvasive measurements of the temporary blood chamber volume in the POLVAD prosthesis with the use of the developed optical system allows us to carry out accurate static and dynamic measurements.

## Background

Heart problems are nowadays the major cause of death. Most heart and cardiovascular diseases are caused by hypertension, which is usually conditioned by the use of drugs. At the same time, we can observe a growing number of patients with III and IV stage heart failures (NYHA scale) [1], in which pressor amine drugs alone can be ineffective and a surgery procedure is required. In such cases, the ventricular assist device (VAD) can be used to support the heart muscle in the pumping process of blood. It releases the heart muscle from much effort, thus the life of the patient waiting for a heart transplant can be prolonged, or in some cases can bring about a partial or full recovery of the heart muscle [2]. There are two main types of VAD used in medical applications: pulsatile and non-pulsatile ones [3]. Recent research studies seem to prove that both types of devices can be successfully used in supporting the heart muscle. Non-pulsatile solutions can be smaller in size and easier to implant in a patient’s body as compared with pulsatile solutions. In spite of that, there are still cases in which pulsatile solutions are preferred [4], especially in the cases when the patient is subjected to conditioning for heart transplantation. The pulsatile solutions are usually paracorporeal, and usually connected with the human ventricular system in parallel with the human heart, thus they can be used in some cases for the recovery of the heart muscle.

### Objective of the research

**Fig. 1:**
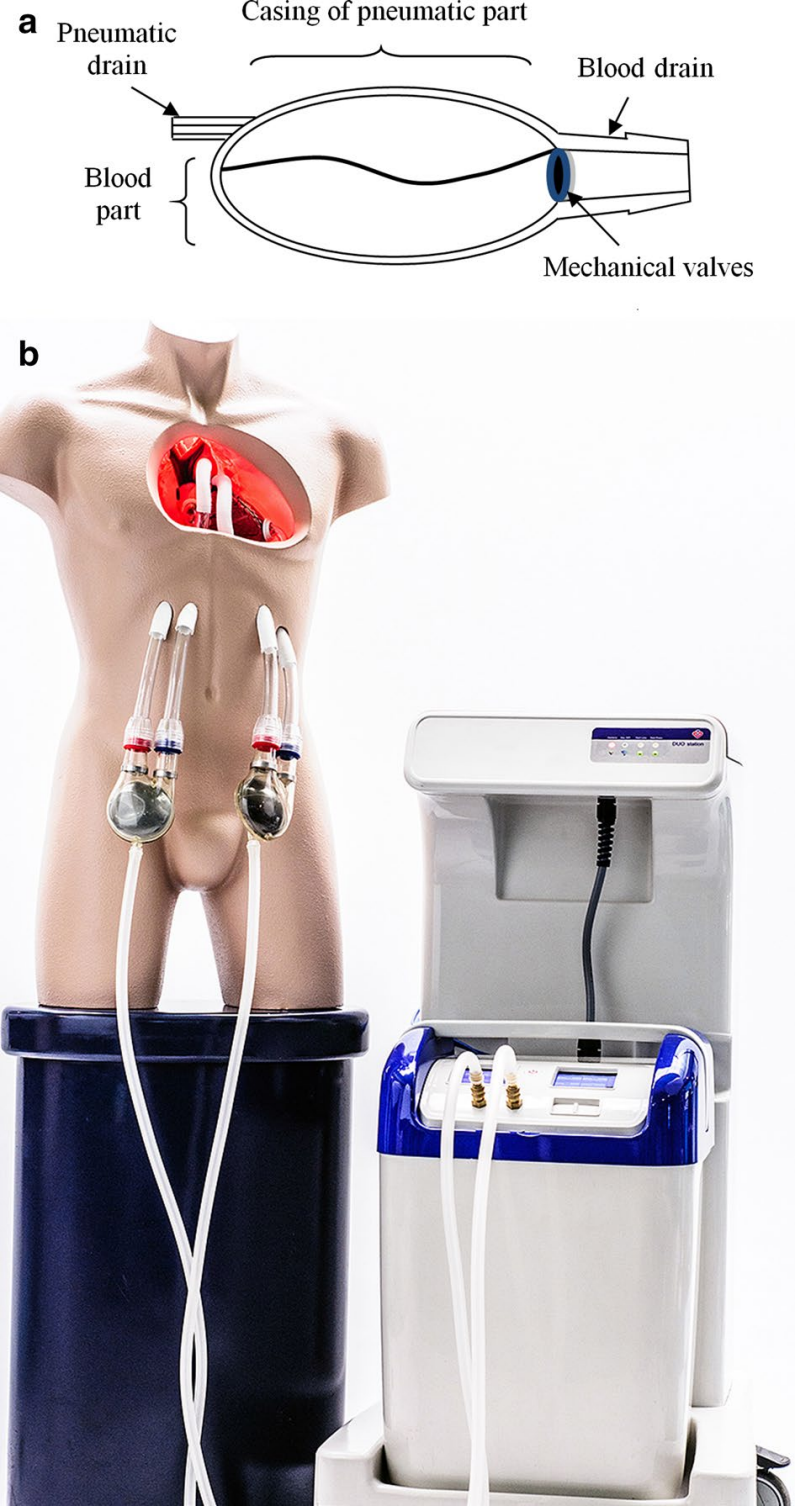
The newly developed RELIGA-EXT prosthesis (construction diagram and the complete heart support system)

The main objective of our research studies is the elaboration of an optical system for measuring blood chamber volume in the paracorporeal pneumatic, pulsatile ventricular assist devices. The experiments involved the modified model of the ReligaHeart EXT (manufactured by the Foundation of Cardiac Surgery Development, Poland). It is a paracorporeal, pulsatile heart support device used in patients for over a decade now. The construction of the POLVAD and ReligaHeart EXT is similar to most solutions of the pulsatile ventricular assist devices utilized in the world (Berlin Heart EXCOR, Medos HIA-VAD, Abiomed BVS 5000 etc.) [3]. The prosthesis consists of two chambers: the blood and pneumatic chambers (separated by a flexible membrane), blood inflow and blood outflow connectors and two mechanical valves. The improved model of the POLVAD, called ReligaHeart EXT, can be seen in Fig. 1. POLVAD and ReligaEeart EXT pumps are clinically utilized in Poland.

The changes of air pressure inside the pneumatic chamber cause the movement of the membrane, thus pumping the blood. Air pressure changes are induced using the pneumatic driving unit (Fig. 1b). The direction of the blood flow is determined by a relative configuration of the valves in the connectors.

### State of art

Cardiac recovery by mechanical heart supporting is a complex process. It occurs in about 20 % of cases involving the mechanical supporting of the heart by means of blood pumps [5]. During the recovery, the cardiac function gradually improves and the total blood flow in the aorta increases [6]. It necessitates a gradual reduction of the mechanical supporting of the heart.

In the current state of art, the POLVAD and ReligaHeart EXT ventricular assist devices, as well as other solutions of such pneumatic assist devices, are not equipped with systems monitoring the actual (transient) blood volume inside the prosthesis. Its semitransparent casing allows the medical staff to evaluate the heart support process visually and by monitoring the patients vitals. This approach requires a continuous medical assistance. Patients need to be hospitalized all the time, which causes a major discomfort and significantly reduces their mobility. The solutions of ultrasound measurements are expensive and large in size. Therefore, their application is mostly limited to clinical conditions.

Polish experience involving the application of the cardiac assist system POLCAS in more than 300 cases proved that the best clinical results were obtained by synchronous supporting [7, 8], commonly with the manual reduction of heart supporting during the recovery. The measurement of the actual blood chamber volume might significantly improve the recovery process by providing feedback information about the cardiac output of the prosthesis. This information about the current state of VAD device would be a milestone in automating the heart support process completely.

Currently, all over the world, the most popular method for estimating the cardiac output of pneumatic VAD is the measurement of flow rate, using the ultrasound flow rate meters [9]. These devices are expensive and fairly large in their size. Although the measurements are accurate, the mobility of the patients is still considerably restricted.

The most important issue in supporting the heart with VAD is to empty completely the device in each cycle, so that the blood might circulate properly in the device. In the case of a partial ejection the volume of blood that remains in the blood chamber has by about 60 % longer contact with the biomaterial than the portion of blood ejected directly from the VAD, which can lead to the formation of thrombus. Moreover, the washing of internal surface of the blood chamber is better in the case of a total ejection than in the case of partial ejection [10, 11]. That is why the determination of the minimum-filling state is of crucial importance.

The existing solutions incorporated in the VAD device and used in patients allow us to determine only the full- and minimum-filling states of the prosthesis, using a simple optical or magnetic switch [12]. This is unfortunately only a partial solution of the problem. In that configuration, the sensor does not allow us to monitor the actual output of the assist device. At some point the air flow measurements in the pneumatic duct were used to measure the pneumatic part volume. The method provided the measurements with the 10–20 % uncertainties [13]. The newer approach to the subject matter was based only on the measurements of pressure [14]. Both methods, however, required frequent calibration cycles, using the reference method. There were many other approaches to solve that problem in the pneumatic type of VAD [15–18]. One of the promising solutions developed in our Department was based on the concept of Helmholtz’s acoustic resonator [17–19]. In that solution, the pneumatic part acts as an acoustic resonator. The elaborated method allows us to determine the actual volume of the prosthesis, but requires an additional sensor chamber over the pneumatic part of the prosthesis. It increases the total volume of the VAD. Although the method was successfully tested in laboratory conditions, it has not been incorporated in the new model of the VAD. To the Authors’ knowledge, there is no solution incorporated in the pneumatic VAD used in patients, allowing to estimate online the blood volume in the VAD device. Another solution developed by the Authors of the manuscript is focused on the application of the ultrasound Doppler method [20]. It was proved that apart from the information about the velocity of the flow, the energy of ultrasound echo contains the information about micro-objects drifting in the blood [21, 22]. This effect offers an opportunity to detect micro-emboli and larger clots, which can be then used in controlling the anticoagulation in order to increase the patient’s safety.

The mentioned acoustic methods had some drawbacks that hindered their application in the prosthesis [17–19]. It motivated the Authors to look for an improved measurement solution.

### Optical measurement system

The experience gained with the acoustic method was used in the development of a new solution—an optical one. The proposed measurement system must be noninvasive to blood environment. That is why, most of transient blood volume measurement methods are taking advantage of the properties of the membrane, which separates the blood chamber and the pneumatic chamber of the VAD, at the pneumatic part. The proposed measurement system has no contact with blood environment. The blood volume in the blood part of the prosthesis is estimated by the measurement of the volume of the pneumatic part.

**Fig. 2:**
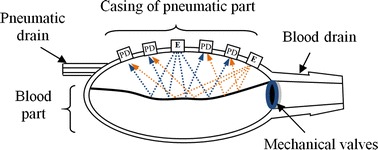
The idea of an optical measurement system with photodiodes (PD) and light emitting diode (E) in the pneumatic part casing

In the new, optical method, several light sources and light detectors are situated in the casing of the pneumatic chamber. The spatial configuration of the light emitting diodes and photodiodes was chosen basing on the previously conducted research studies at the Department of Optoelectronics. The light emitted by light emitting diodes (E), after direct reflection on the membrane at the pneumatic side, is detected by photodiodes (PD). The principle of the optical measurement system can be seen in Fig. 2. For each volume of the pneumatic part of the VAD, all the related amplitude signals on the photodiodes, for all configurations of the light emitting diode–photodiode, are measured. The signal is then analyzed. Since the position of the membrane changes, the amplitudes of the detected signal for some of light emitting diode–photodiode pairs also change, and thus the actual blood chamber volume can be estimated. During the process of blood pumping the shape of the membranes surface changes in a random manner. That is why reflectance measurements based on a single sensor element alone [23, 24] cannot be used. In preliminary research studies of the optical measurement system the membrane reflectance properties were extensively examined, using the spectral ellipsometry (Sentech SE850 ellipsometer) within the range 400–1000 nm, in order to find the optimal range of the wavelengths of light that could be utilized in the system [25]. It turned out that the maximum reflectance occurred at the UV range, but the difference of energy reflectance was less than 10 % in the examined spectrum range, with the minimum value at 770 nm. Due to the wide selection of light emitting diode and photodiode elements, as well as the availability of photodiodes with high-pass filters, allowing to immunize the optic system to visible light, it was decided that the IR range should be used. In order to minimize the effect of the shape of the membrane on the measurement results, wide-angle light emitters and light detectors were used. The complimentary pair of light emitting diode (SFH203FA) and photodiode (SFH203PFA) manufactured by Osram, operating at peak wavelength of 880 nm, was used in the final solution [26]. The matrix of light emitters and light detectors used in the research studies was incorporated in the pneumatic part of the casing (Fig. 2).

The VAD model used in the performed research studies was produced using rapid prototyping techniques. The internal dimensions of the model are the same as in the medically used VADs.

The membrane which is used to separate the blood part from the pneumatic part is exactly the same as that used in actual prosthesis: two layers made of *Chronoflex ARLT*, separated by the graphite powder for reducing the friction between the two layers.

### Preliminary research studies

The preliminary research studies (performed at the Department of Optoelectronics, Silesian University of Technology, Poland) comprised static and low dynamicity testing (<5 ml/s), aiming to determine the behavior of the membrane and the development of the electronics and software for the measurement system [27, 28]. Since the membrane in this construction changes its shape in a random manner during the process of blood pumping, it was decided that in the first stage of the investigations, the optical method would use 12 light emitting diodes and 32 photodiodes. In each measurement cycle, each IR light emitting diode was powered (one at a time), and the signal from all 32 photodiodes was detected. In order to achieve the sufficient speed of acquisition, fast multiple input A/D converters had to be used, along with precise amplifiers connected to each of the photodiodes. Four 8-channel A/D converters were used, interrogated by 2 MHz SPI bus. The proper time delay between the consecutive interrogations introduced by the software, was based on research studies concerning the photodiode circuit response times, after the light emitting diodes had been lit. The time of one cycle for all single light emitting diode–single photodiode configurations (equal to 384) amounted to 100 ms.

**Fig. 3:**
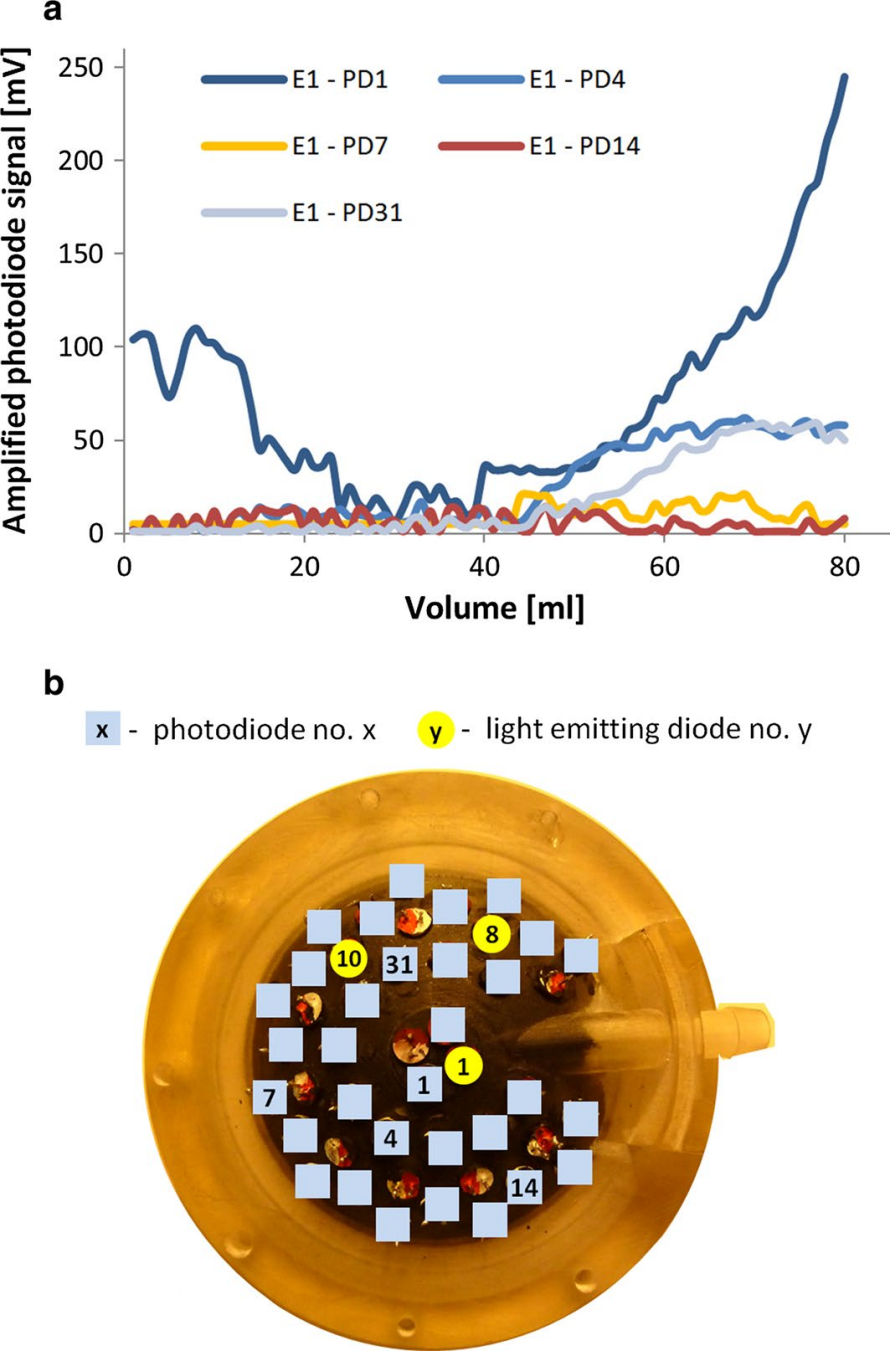
Relation between the actual volume and signals from each of the 32 photodiodes (**a**), when only light emitting diode E1 is used (**b**)

The relations between the volume and amplitude on some photodiode combinations for diode 1 are shown in Fig. 3a. The arrangement of the photodiode and the light emitting diode in the pneumatic casing can be seen in Fig. 3b. Some of the pair signals depended on the changing volume of the pneumatic part in the whole range of the volumes (e.g. E1-PD1), and some combinations could provide information about the volume in the limited range (e.g. E1-PD31, E1-PD4). Some of the E-PD combinations could not provide any meaningful information (e.g. E1-PD7, E1-PD14). It turned out that the direct analysis of data could not be used—none of the single light emitting diode–single photodiode combinations could provide direct information about the pneumatic chamber volume.

Due to the above and because of the significant quantity of data to be processed, it was decided that data-mining techniques should be used.

The PCA (principal components analysis) method was used, mainly because of its nonparametric operation, the possibility to reduce the number of dimensions of the input data, as well as to identify of redundant data [26]. The measurement results were then classified basing on the volume of the blood part, measured using the reference method. The process was repeated for different objective functions, aiming at the best separation of the data points for different blood part volumes. This process resulted in a 3D space, formed by three first principal components, where selected blood part volumes were represented (each 5 ml in a range of 0–80 ml). The process of finding the actual volume required the transformation of the input data into the 3D space of the principal components, and the localization of the nearest volume using k-NN (k Nearest Neighbors) algorithm. The algorithm allowed us to determine unequivocally the volume of the pneumatic part, when three adjacent neighbors were localized.

The analysis resulted in the selection of a set of three light emitting diodes (E1, E8, E10) (marked in Fig. 3b) and some photodiodes, allowing to reduce the required number of configurations (features) to 34. The calculated function allowed us to determine the volume in low dynamic conditions [26]. A more detailed description of this problem can be found in [27].

The successful preliminary results obtained by means of PCA and k-NN [26, 27] provided a solid basis, allowing to prepare the optical measurement system for dynamic measurements at the Foundation of Cardiac Surgery Development (FRK). The system was modified to acquire measurement results with the required rate. The measuring time of a single cycle was reduced from 100 to 35 ms for all combinations (384 E-PD pairs). Additionally, the configurations of the chosen light emitting diodes and photodiodes were programmed on the basis of the data acquired in the course of the static measurements. It enabled a further increase of the acquisition speed, resulting in 10 ms measurement cycles for the selected combinations.

## Methods

Preliminary dynamic measurements of the optical measurement system were performed at the FRK. The physical model of the human cardiovascular system was used [28]. The independent volume measurement method consisting of two ultrasound flow rate meters (at the inflow and outflow of the prosthesis) was used as the reference. Transonic T410 (11PLX transducer—5 % uncertainty) flow rate meters were applied in the experiment. The ultrasound sensors used in the measurements were previously calibrated using the Fluxus ultrasound flowmeter (2 % uncertainty).

The blood-like liquid used during the measurements was a 60 % water solution of glycerin mixed with benzoic aldehyde (capillary viscometer measurement result: 8.5 cP at 25 °C).

**Fig. 4:**
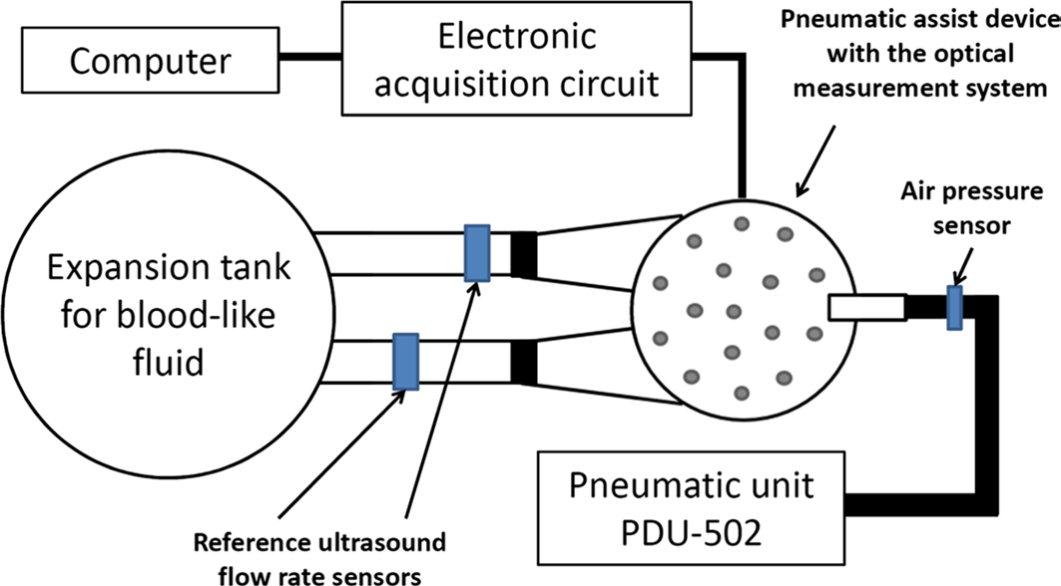
The idea of the measurement stand at the Foundation of Cardiac Surgery Development, Zabrze, Poland

The synchronization of the time of volume measurements obtained by the application of the developed optical system and volume measurements derived from the flow rate was obtained using the pressure signal in the pneumatic drain. The pressure signal was measured simultaneously in both measurement stands. In the case of dynamic tests, the prosthesis was controlled by the pneumatic driving unit PDU-502 (produced by the FRK), utilized in clinical trials. The concept of the measurement stand used in the testing can be seen in Fig. 4.

During the tests, several heart support speeds (average heart rate—AHR) and blood duct impedances were used. This allowed us to analyze various working modes of the prosthesis: full filling/full ejecting, full filling/partial ejecting, partial filling/partial ejecting and partial filling/full ejecting.

## Results and discussion

**Fig. 5:**
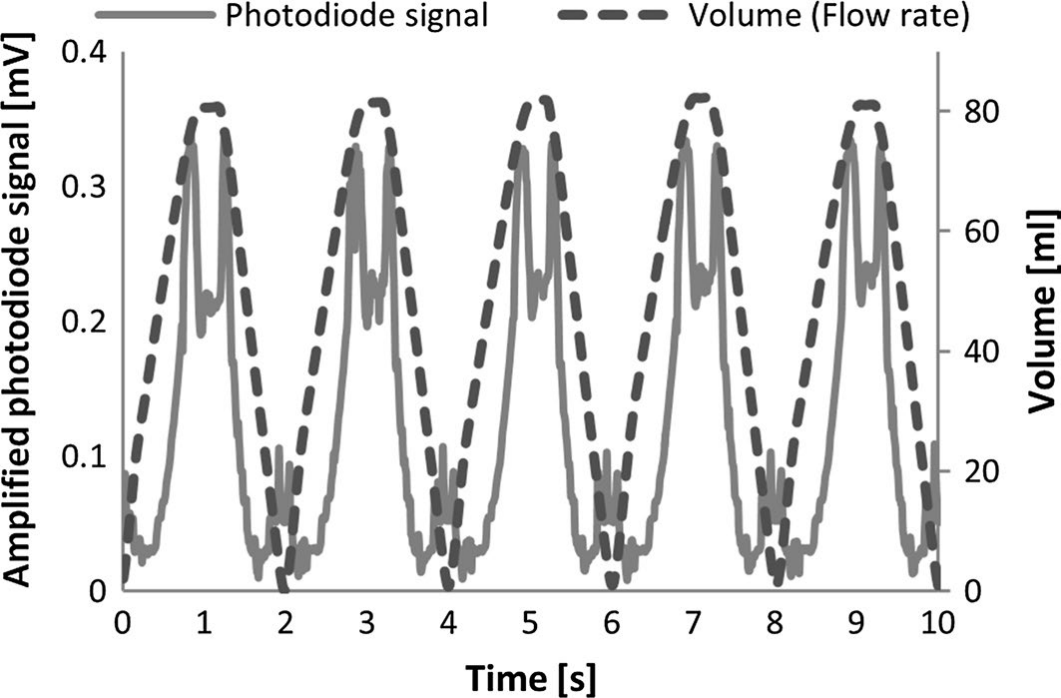
Volume resulting from the flow rate and an exemplary optical signal amplitude (light emitting diode1–photodiode 1) characteristics in time

The results were the same as in the case of previous research studies with low dynamics [25, 26]. The photodiode signal amplitudes were repeatable with respect to the blood chamber volume characteristics over the whole range of working conditions of the prosthesis. Figure 5 shows the exemplary characteristic of a chosen photodiode signal in the time domain, accompanied by the volume derived from the flow rate.

**Fig. 6:**
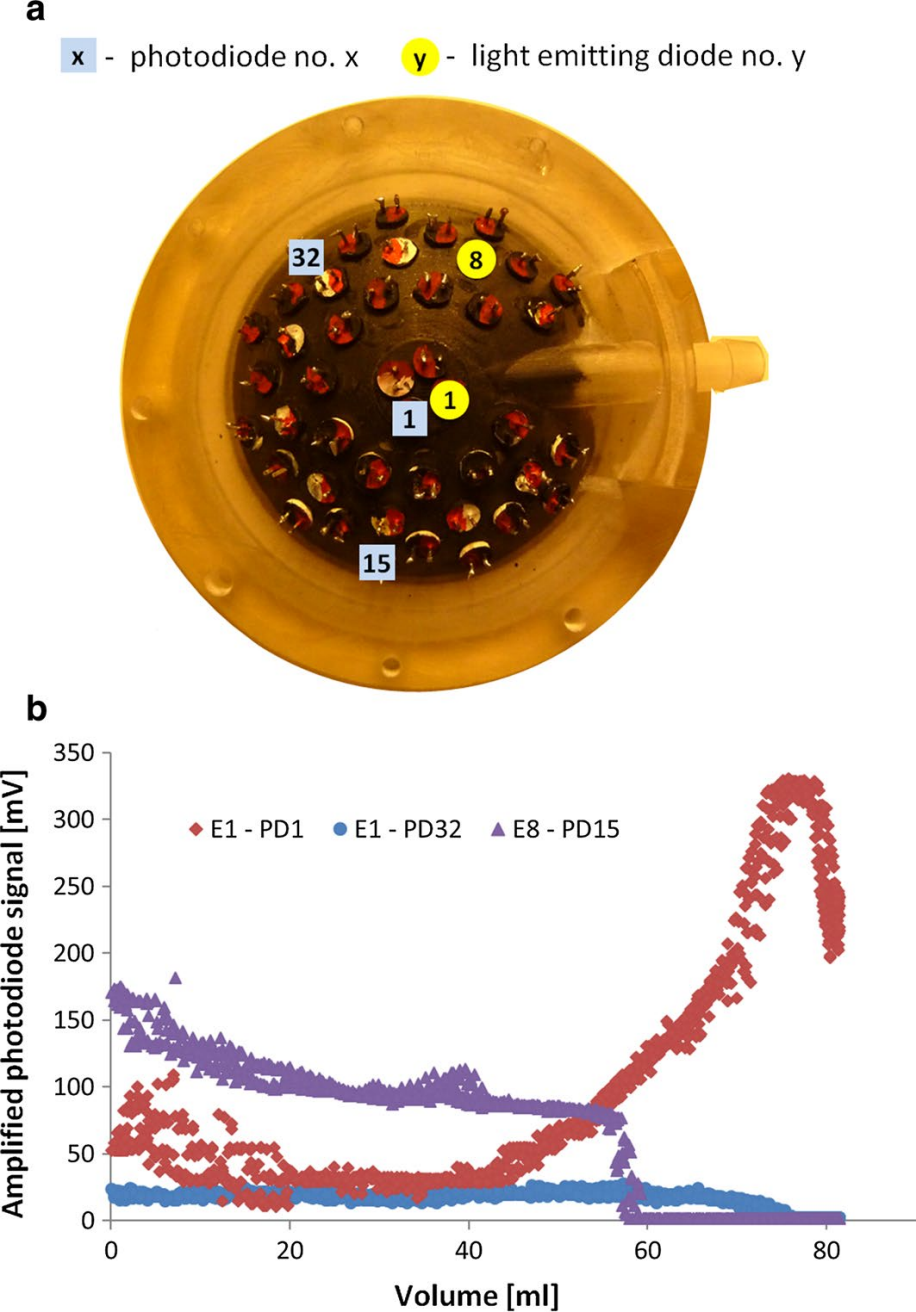
The selected light emitting diode-photodiode configurations (**a**) and the relating photodiode signals as a function of the volume of the blood part (**b**)

The photodiode signal shown in Fig. 5 was achieved by using the centrally localized light emitting diode and photodiode shown in Fig. 6a (light emitting diode 1–photodiode 1). Figure 5 shows the amplitude of the detected signal for all of the volumes of the blood part of the VAD. It can be seen that the characteristic is equivocal. When the membrane reaches the casing of the pneumatic part, the light emitting diode and the photodiode are partially covered by the membrane—this results in two maximum peaks in this area. At very low volumes the membrane starts acting as a concave mirror structure, resulting in a slight increase of the detected amplitude for this specific configurations.

Dynamic measurements were realized under various driving pressures and various blood duct impedances at the inflow and outflow cannulas, which allowed us to modify the filling and ejecting process of the prosthesis under similar driving pressures.

The complete set of optical measurements and the ultrasound measurements of the derived flow rate data was analyzed by means of the numerical methods (PCA and k-NN).

The PCA was used to select the light emitting diode–photodiode configurations that could provide an unequivocal relation between the amplitude on the detectors and the volume of the blood part.

During the dynamic tests, realized on the model of human cardiovascular system, the elaborated system acquired data from all 384 configurations. Additionally, the reduced number of configurations (34 features preselected by the PCA) was used in a faster acquisition program.

Due to the requirement of limited changes in the construction of the prosthesis, inflicted by the manufactures of the cardiac support system, in order to maintain the integrity of the construction of the prosthesis casing, it was decided that the number of sensor elements should be limited. Basing on the manufacturers’ suggestions, in order not to hinder the mechanical properties of the casing of the prosthesis, it was agreed that the maximum number of light emitting diode and photodiode elements incorporated into the casing of the prosthesis should not exceed eight.

### Measurement results

The Authors were able to select three configurations out of 34 preselected previously using the PCA, that provided the possibility to determine an unequivocal transfer function.

The combinations that were used in the final solution are: E1-PD1, E1-PD32, E8-PD15 (Fig. 6a).

Basing on the amplitude of the signal on the photodiode for the particular configurations, it is possible to determine the volume of the blood part. The configuration E8-PD15 is used to determine the lower volumes (<35 ml); E1-PD1 is used for the range 35–75 ml. The E1-PD32 configuration is used to detect the equivocal part of the E1-PD1 configuration at higher volumes (>75 ml) (Fig. 6b).

**Fig. 7:**
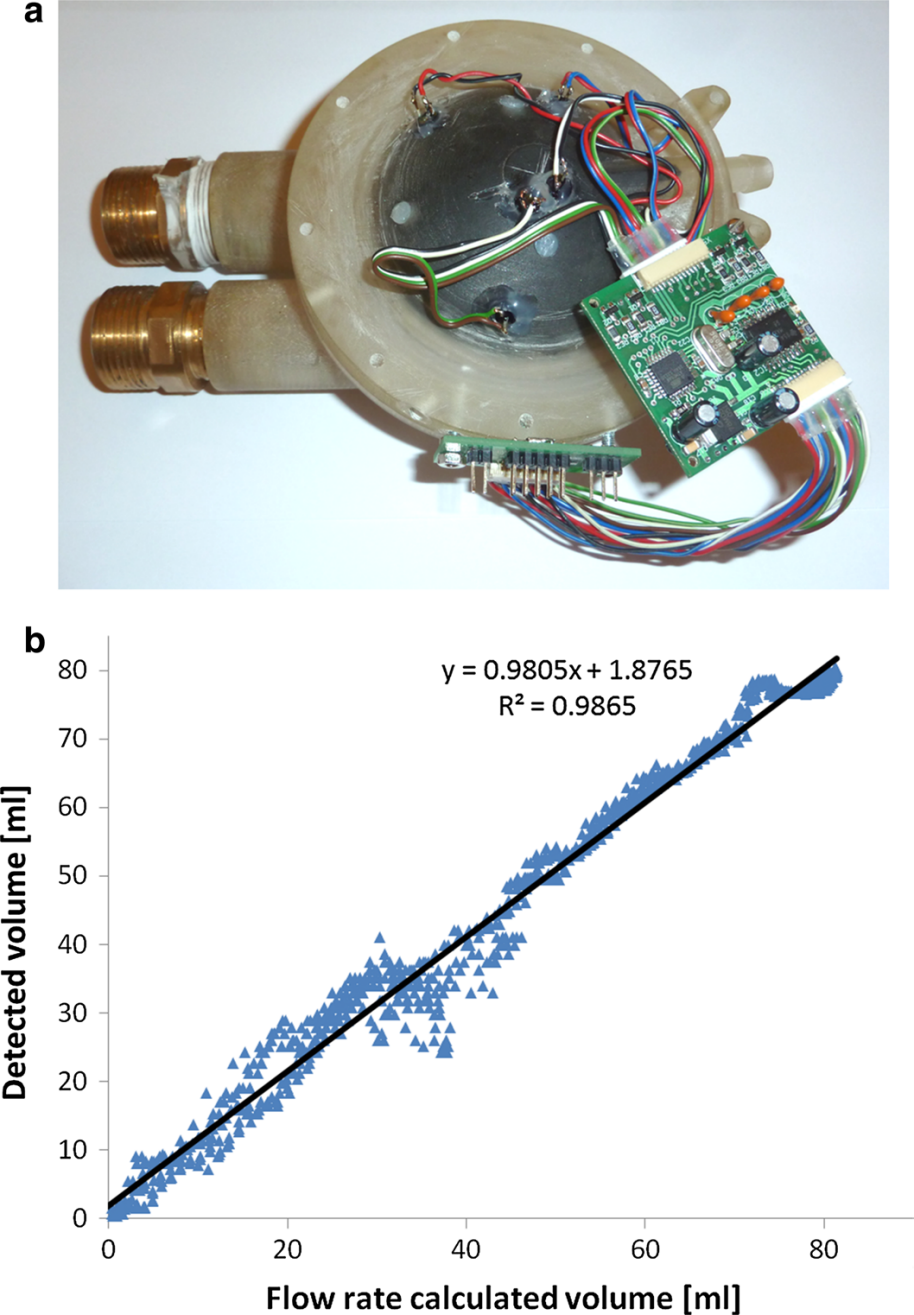
The reduced optical measurement system (**a**), and the relation between the volume detected by the optical system and derived using the flow rate based measurement system (**b**)

**Fig. 8:**
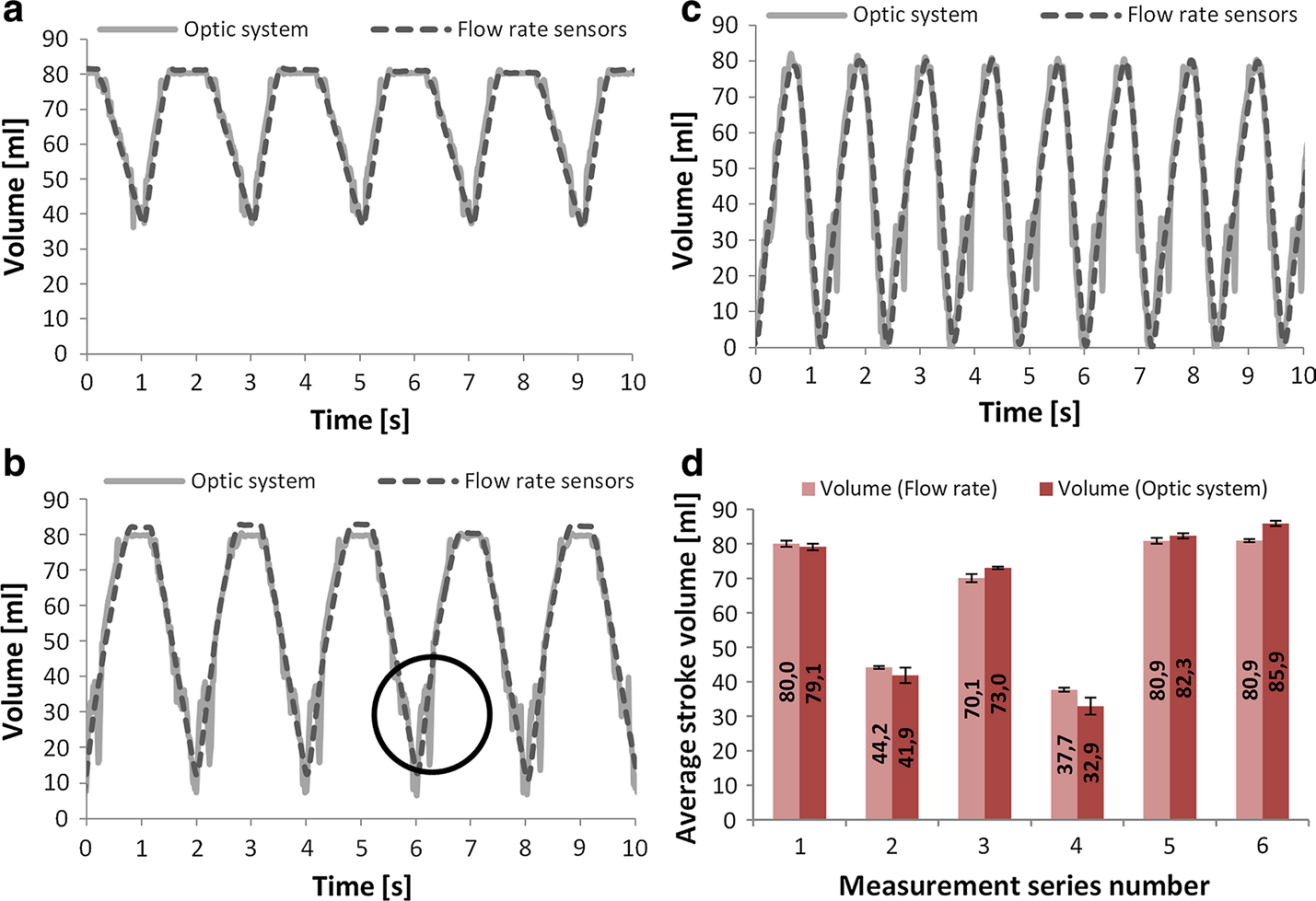
Exemplary results of dynamic tests carried out on the physical model of the human circulatory system in various conditions of heart support in the time domain (**a**, **b** AHR = 30, **c** AHR = 50) and comparison of the results of measurements acquired using the optical system and the system based on flow rate sensors (**d**)

All of the configurations turned out to behave differently in the filling and emptying cycle, otherwise being repeatable in all cycles. This dependence is visible in mid-range volumes (30–40 ml) (Fig. 7b). This results in the artefacts marked in Fig. 8b.

Knowing the required number of configurations, the size of the measurement system was reduced; the electronic circuit was redesigned to a more compact one. In order to reduce the size of the electronic circuit, a 4-layer transducer board was used. In Fig. 7a the reduced preliminary system is shown. Finally, the optical system was reduced to two light emitting diodes (E1, E8) and three photodiodes (PD1, PD15, PD32) marked in Fig. 6a.

Figure 8 presents the volume calculated with the use of flow rate measurements and the volume results from the optical system.

We can observe in Fig. 8a–c that the blood volume measurements obtained by means of the new optic method coincide with the results calculated with the use of flow rate measurements. The exemplary results show three different test series: 8a—operating with AHR = 30, at approximately half of the maximum stroke volume of the VAD; 8b—operating with the same AHR and pneumatic driving pressures, but with slightly reduced blood duct impedances resulting in larger stroke volume of the VAD; 8c—VAD operating at AHR = 50 bpm with full stroke volume). The measurements of mid-range (30–40 ml) volumes showed the artefacts marked in Fig. 8b. They can be related to the rapid change in the shape of the membrane (from convex to concave character in relation to the pneumatic part casing) and ought to be improved. When the impedances of the blood duct change, which takes place in the case of the human cardiovascular system, the same driving pressures of the VAD result in a different stroke volume (Fig. 8a, b). By comparing the driving pressures with the volumes of the blood part acquired using the proposed method, it would be possible to detect the changes in the impedance of the blood duct, providing important information concerning the state of the supported cardiovascular system.

In Fig. 8d, we compared the average SV (stroke volume) results obtained in the optic system with average SV results obtained using the flow rate measurements at five experiments under different heart support conditions. The stroke volume measurements can be used for the estimation of the CO (cardiac output) of the VAD, which is a well-recognized parameter in heart support processes.

The redesigned solution will be a subject of extended tests in the presence of clinically applied ultrasound flow rate sensors as a reference method of measuring the volume.

## Conclusions

The paper presents the results of investigation studies involving dynamic blood chamber volume measurements of the pulsatile, pneumatic type heart support device. The research studies are focused on the development of an optical measurement system. The dynamic measurements were performed on the model of the human cardiovascular system at the Foundation of Cardiac Surgery Development in Zabrze, Poland [28]. Ultrasound flow rate sensors were used as a reference volume measurement method. The method was tested in changing operating conditions of the VAD device with various driving pressures of the heart assist device, various impedances in the blood duct, varying heart rates.

The results of the performed measurements, making use of the optical system and the reference method, show that the proposed optical method can be applied for accurate blood chamber volume measurements. A direct comparison of the average blood output volume during consecutive cycles using both methods confirms the above declaration.

Future research studies will comprise the examination of a miniaturized measurement system as well as an improvement of the measurement algorithm. The optical measurement system meets all requirements for a noninvasive transient blood volume measurement system.

The proposed method is designed for pulsatile type VADs, which are usually extracorporeal solutions. In that case, the device will be directly powered. The elaborated system at the current state of development requires approximately 100 mW of electric power. Further investigations aiming at miniaturizing the measurement system should provide an additional essential reduction of power consumption, which would be important if used in the future in intracorporeal pulsatile VADs, or battery powered solutions.

The proposed optical solution of transient blood volume measurements has overcome the issues existing in measurement systems based on the acoustic method [17, 18].

According to an extended search in literature and the Authors’ knowledge, the proposed measurement method (and the elaborated system) is an original and novel solution of blood chamber volume measurements in pneumatic heart assist devices. The optical solution has been submitted to be patented [29].

The optical method of blood volume measurement presented in the paper may be used as a method to assess the location of the membrane (e.g. to detect the full filling and full ejection of blood from the blood chamber). In conjunction with an ultrasound Doppler assessment of micro-emboli it may constitute an automated extracorporeal cardiac assist system.
